# Diabetes prediction, lipid accumulation product, and adiposity measures; 6-year follow-up: Tehran lipid and glucose study

**DOI:** 10.1186/1476-511X-9-45

**Published:** 2010-05-10

**Authors:** Mohammadreza Bozorgmanesh, Farzad Hadaegh, Fereidoun Azizi

**Affiliations:** 1 Prevention of metabolic disorders research center, Research institute for endocrine sciences (RIES), Shahid Beheshti university (M.C.), Tehran, Iran

## Abstract

**Background:**

The body mass index (BMI) is the most commonly used marker for evaluating obesity related risks, however, central obesity measures have been proposed to be more informative. Lipid accumulation product (LAP) is an alternative continuous index of lipid accumulation, which is computed from waist circumference (WC, cm) and triglycerides (TGs, mmol/l): (WC-65) ×TG (men) and (WC-58) ×TG (women). We sought in this study to assess if LAP can outperform BMI, waist-to-height-ratio (WHtR), or waist-to-hip-ratio (WHpR) in identifying prevalent and predicting incident diabetes.

**Results:**

The cross-sectional analyses were performed on a sample included 3,682 men and 4,989 women who were not pregnant, aged ≥ 20 years. According to the age (≥ 50 and <50 years) - and sex-specific analyses, odds ratios (ORs) of LAP for prevalent diabetes were higher than those of BMI, WHpR, or WHtR among women, after adjustment for mean arterial pressure and family history of diabetes. The OR of LAP in old men was lower than those of other adiposity measures; in young men, however, LAP was superior to BMI but identical to WHpR and WHtR in identifying prevalent diabetes. Except in young men, LAP showed highest area under the receiver operating characteristic curves (AROC) for prevalent diabetes (P for trend ≤ 0.005).

For longitudinal analyses, a total of 5,018 non-diabetic subjects were followed for ~6 years. The ORs of BMI, WHpR, and WHtR were the same as those of LAP in both sexes and across age groups; except in young men where LAP was superior to the BMI. AROCs of LAP were relatively the same as anthropometric adiposity measures.

**Conclusions:**

LAP was a strong predictor of diabetes and in young individuals had better predictability than did BMI; it was, however, similar to WHpR and WHtR in prediction of incident diabetes.

## Background

There is an emerging view that type 2 diabetes (hereafter diabetes) may reflect not so much as an isolated impairment of glucose regulation, rather the complex metabolic consequences of accumulating ectopic lipids or hepatic fat [1-4]. Obesity is commonly used to imply excess fat, but it is ordinarily classified according to excess weight. This semantic inconsistency may help to explain why the body mass index (BMI) performs only modestly as a predictor of medical risk [5].

Lipid accumulation should be defined and measured specifically in those contexts where accumulation may represent a physiologic danger [6,7]. These contexts might be described as lipid over accumulation [8]. Attributing culpability to components of adipose or lean tissue should also be avoided; since although enlarged, they might enhance physiologic processes or reduce the risk of disease.

Kahn described a simple index for estimating lipid over accumulation among adults - designated the "lipid accumulation product" (LAP) - based on a combination of two measurements that are safe and inexpensive to obtain. One is waist circumference (WC), a measure of truncal fat that includes the visceral (intra-abdominal) depot. The other is the fasting concentration of circulating triglycerides (TGs). The simple index described there was developed to express a continuous risk function. If LAP is better correlated than BMI with cardiovascular risk factors, this finding would support the notion that the over accumulation of lipid carries worse cardiovascular consequences than the less specific over accumulation of weight [9].

In a population based cross sectional study LAP has been shown to be better than BMI in identifying prevalent diabetes [1]. The BMI can neither distinguish between fat and lean tissues nor identify the anatomic location or function of distinct fat depots. But abdominal adiposity has been shown to be more closely linked with adverse metabolic consequences and has been suggested to precede insulin resistance [9].

An unanswered question in original description of LAP was if LAP could be a strong predictive of diabetes. Less is known concerning the performance of LAP as compared to the measures of abdominal adiposity. Our primary focus in this study, therefore, was to assess if LAP can outperform BMI, waist-to-height ratio (WHtR), or waist-to-hip ratio (WHpR) in identifying prevalent- or predicting incident-diabetes.

## Methods

### Study design and population

Between 1999 and 2001 the Tehran Lipid and Glucose Study (TLGS) recruited a population-based cohort of more than 15,000 residents aged over 3 years from district 13 of Tehran, as a representative of Tehran population to participate in a baseline study, of whom 10,368 aged ≥ 20 years. The rationale and design of the study has been described elsewhere [10].

**Figure 1:**
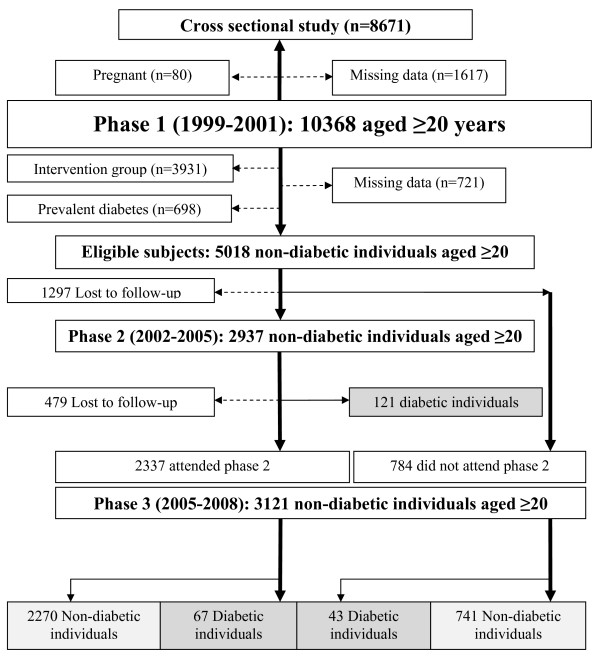
**Study population: included, excluded, lost to the follow-up and events, and censored individuals**. Starting point is black text-box with upward, for cross sectional study; downward, for longitudinal study.

For longitudinal analyses, we excluded individuals assigned to the intervention study (n = 3,931), those with prevalent diabetes mellitus (using oral hypoglycemic agents or insulin, baseline fasting plasma glucose (FPG) ≥ 7.0 mmol/l or 2 hour post challenge plasma glucose (2h-PCPG) ≥ 11.1 mmol/l, n = 698), and those with incomplete data on their diabetes status (n = 623) or baseline clinical measurements (n = 98). The remaining 5,018 non-diabetic subjects were followed up to the second (2002-2005) and third (2005-2008) TLGS examinations (end point for these analyses), ~3 years apart, for an average period of ~6 years (Figure 1). The screening method used at the baseline as well as the follow-up visits was the same and included measurements of both FPG and 2h-PCPG. Participants who left the study before the end point for the longitudinal analyses or before first developing diabetes were excluded. The final sample for longitudinal analyses consisted of 3,242 participant. The main reasons for lack of attendance at follow-up examinations, despite repeated calls, were either migration or other personal reasons. Informed written consent was obtained from all subjects and the Ethical Committee of Research Institute for Endocrine Sciences approved this study.

The cross sectional analyses was performed on a sample included 3682 men and 4989 women, aged 20 years and over, who were not pregnant, with all relevant data available (Figure 1).

### Clinical, anthropometric, and laboratory measurements

The baseline examination obtained information on family history of diabetes and medication use. The standard 2 hour post-challenge plasma glucose (2h-PCPG) test was performed for all individuals ≥ 20 years, not on glucose lowering drugs. Height, weight, WC, plasma glucose level, and serum triglycerides (TGs) levels were measured using previously reported methods [11]. BMI was calculated as weight in kilograms divided by height in meters squared. WHpR was calculated as waist circumference (WC) divided by hip circumference and WHtR was calculated as WC divided by height.

### Definition of variables and outcomes

Positive family history of diabetes was defined as having at least one parent or sibling with diabetes. Participants were classified as having developed new diabetes during follow-up if they met at least one of these criteria: FPG ≥ 7 mmol/l, or 2h-PCPG ≥ 11.1 mmol/l or taking anti-diabetic medication. Impaired fasting plasma (IFG) and Impaired glucose tolerance (IGT) were defined as FPG from 5.6-6.9 mmol/l and 2h-PCPG from 7.8-11.0 mmol/l, respectively [12]. The LAP was defined to, theoretically, describe the extent to which an individual had travelled the route of both increasing waist and increasing TG [9]:

Mean arterial pressure (MAP) was calculated as:

### Statistical analysis

All mean are presented as mean (standard deviation). The mean values and the proportions of baseline variables across quartiles of LAP were compared, in each sex, using the linear and logistic regression model, respectively. To identify whether LAP performs better than the anthropometric variables, i.e. BMI, WHpR, or WHtR for identifying elevated FPG, 2h-PCPG, the linear regression model was applied. Sex- and age-specific (20-49, ≥ 50 years) estimates of FPG and 2h-PCPG based on LAP, BMI, WHpR, or WHtR, were derived from linear regression models. Baseline variables (LAP, BMI, WHpR, WHtR, FPG, and 2h-PCPG) were log_e_-transformed before standardization to improve discrimination and calibration of the models and to minimize the effect of extreme observations [13]. The correlation coefficient (R) represents the linear relationship between two continuous variables. The squared correlation coefficient (R^2^), generally known as the coefficient of determination, represents the proportion of common variation in the two variables i.e. the strength or magnitude of the relationship. The LAP and BMI, WHpR and WHtR were compared in regards of the proportion of the total variation in FPG and 2h-PCPG, that each index could explain, which is R^2 ^for each regression model. Logistic regression was used to assess contribution of each risk factor to the risk of prevalent diabetes IFG, IGT and incident diabetes. Wald tests of the linear hypotheses concerning the logistic regression models coefficients (paired homogeneity test) were performed to test the null hypotheses that the odds ratios (ORs) (effect size) for LAP were equal to those for BMI, WHpR, or WHtR. As the anthropometric measurements were highly correlated, the ORs obtained from logistic regression models including more than one measure were difficult to interpret because of collinearity. Because BMI, WHpR, WHtR, and LAP were highly correlated, we assessed collinearity between these variables using condition indices and variance inflation factor (VIF). Condition indices >30 or VIFs >10 warrant caution [14]. Effort was made to reduce collinearity by modeling variables in quartiles and standardizing the log_e_-transformed variables. Receiver operating characteristic (ROC) curve analysis [15] was used to determine, in combination with family history of diabetes, and MAP, whether LAP would better discriminate incident diabetes cases than did other anthropometric variables. The area under the ROC curve (AROC) from the prediction of multivariate-adjusted LAP was compared with the AROCs from the prediction of multivariate-adjusted models including anthropometric variables (each investigated one at a time). The AROCs were compared according to the method suggested by DeLong [16]. We reported P value without adjusting for pairwise multiple comparisons. Reporting such a P value will allow each reader to decide whether the reported differences are important or not [17,18].

## Results

A total of 8,671 and 3,242 participants, with 42.8% and 42.2% being male, included in the cross sectional and longitudinal study, respectively. Mean age of the subjects was 42.9 ± 15.0 and 41.6 ± 13.2 in cross sectional and prospective study respectively.

Table 1 represents baseline characteristics across LAP quartiles by sexes. Cut-points for LAP were higher in women than in men. In both sexes the prevalence of family history of diabetes, diabetes, IFG, and IGT as well as mean levels of systolic and diastolic blood pressure, MAP, WHpR, WHtR, BMI, age, FPG, and 2 h-PCGP at the time of the LAP measurement progressively increased across LAP quartiles.

**Table 1:** Baseline characteristics of participants in longitudinal study.

Variable	Q1 LAP	Q2 LAP	Q3 LAP	Q4 LAP	P for trend
Men					
LAP	0.62-21.96	21.97-40.93	40.94-68.51	68.52-570.26	
Age	33.1(15.4)	43.3(13.5)	45.0(13.3)	44.7(12.6)	<0.001
FHx of DM	67(20.1)	75(22.5)	94(28.2)	97(29.1)	= 0.002
DM	8(9.1)	13(14.8)	29(33.0)	38(43.2)	<0.001
IFG	19(9.)	747(24.0)	48(24.5)	82(41.8)	<0.001
IGT	14(0.9)	34(21.9)	44(28.4)	63(40.6)	<0.001
SBP	114.3(16.6)	117.3(14.9)	122.6(18.4)	124.7(16.6)	<0.001
DBP	73.3(9.8)	76.8(9.3)	80.4(10.4)	82.1(10.1)	<0.001
MAP	87.3(10.7)	90.4(10.2)	94.4(12.0)	96.3(11.2)	<0.001
BMI	21.6(2.4)	25.0(2.3)	27.1(2.7)	29.0(3.4)	<0.001
WHpR	84.0(4.8)	90.3(4.7)	94.3(5.0)	96.7(5.1)	<0.001
WHtR	44.5(4.1)	50.1(3.9)	54.6(4.2)	57.7(5.1)	<0.001
FPG	4.9(0.5)	5.1(0.5)	5.1(0.5)	5.2(0.6)	<0.001
2h-PCPG	5.0(1.4)	5.6(1.6)	5.9(1.7)	6.2(1.8)	<0.001
Women					
LAP	0.56-22.26	22.27-42.90	42.91-73.98	73.99-620.39	
Age	31.2(8.5)	39.6(11.5)	43.9(11.9)	50.0(11.1)	<0.001
FHx of DM	103(19.4)	135(25.4)	140(26.4)	153(28.8)	<0.001
DM	5(3.5)	19(13.3)	48(33.6)	71(49.7)	<0.001
IFG	14(6.20)	43(19.1)	72(32.0)	96(42.7)	<0.001
IGT	14(5.1)	42(15.2)	86(31.2)	134(48.6)	<0.001
SBP	106.8(10.9)	114.3(16.1)	120.2(16.7)	125.7(19.3)	<0.001
DBP	71.3(8.2)	76.4(9.5)	79.3(9.4)	82.2(9.5)	<0.001
MAP	83.1(8.2)	89.0(10.8)	92.9(10.8)	96.7(11.9)	<0.001
BMI	23.0(3.2)	26.8(3.4)	29.2(3.7)	31.5(4.3)	<0.001
WHpR	75.6(5.1)	81.8(6.2)	86.1(6.1)	89.5(6.5)	<0.001
WHtR	46.4(4.5)	53.8(5.2)	58.8(5.3)	63.5(6.2)	<0.001
FPG	4.7(0.4)	4.9(0.5)	5.1(0.5)	5.2(0.6)	<0.001
2h-PCPG	5.2(1.2)	5.9(1.4)	6.4(1.5)	6.9(1.6)	<0.001

All continuous variables are represented as mean (SD) and all dichotomous variables as number (percent). P values for trends were obtained from linear or logistic regression models for continuously- and binary-distributed variables, respectively.

BMI, body mass index; DBP, diastolic blood pressure; DM, diabetes mellitus; FHx, family history; PFG, fasting plasma glucose; IFG, impaired fasting glucose; IGT, impaired glucose tolerance; LAP, lipid accumulation product; MAP, mean arterial pressure; Q, quartile; SBP, systolic blood pressure; 2 h-PCGP, 2-hour post challenge plasma glucose; WHpR waist to hip ratio; WHtR, waist to height ratio.

### Baseline and future plasma glucose levels prediction

As shown in Tables 2, LAP had almost consistently stronger association (higher R^2^) with baseline and future levels of FPG and 2h-PCPG than did BMI. In women and old men, the LAP explained greater variability in the baseline levels of FPG and 2h-PCPG than did WHpR and WHtR. In young men, however, the amount of variability in the baseline levels of FPG and 2h-PCPG explained by WHpR was higher than those of other predictors. The variations in the future levels of FPG were best explained by LAP in young men and women. Among older men and women, the variations in the future levels of FPG were best explained by BMI and WHtR, respectively. In young women, the variations in the future levels of 2h-PCPG were best explained by WHtR. In younger participants, the variations in the baseline and future levels of FPG and 2h-PCPG were generally better explained by baseline LAP, BMI, WHpR, and WHtR than it were in older ones.

**Table 2:** Percentages of the variation* (adjusted R^2^) in 2h-PCPG and FPG explained by LAP, BMI, WHtR, and WHpR.

	FPG		2h-PCPG	
	**Cross-sectional**	**Longitudinal**	**Cross-sectional**	**Longitudinal**

Male				
20-49				
LAP	5.8	8.40	10.1	7.80
BMI	3.6	6.90	5.7	5.40
WHpR	5.9	7.10	11.5	8.60
WHtR	5.3	8.30	10.2	8.60
≥ 50				
LAP	5.6	2.20	8.8	5.00
BMI	3.2	2.70	4.9	6.00
WHpR	3.2	2.20	6.3	8.00
WHtR	3.6	2.10	6.5	10.20
Female				
20-49				
LAP	10.2	7.80	17.3	10.40
BMI	6.9	5.10	9.8	5.80
WHpR	6.8	5.00	10.5	6.00
WHtR	9.3	6.10	13.6	7.40
≥ 50				
LAP	7.2	3.50	8.5	7.30
BMI	2.4	3.10	1.2	3.80
WHpR	3.6	3.70	5.1	7.10
WHtR	4.6	4.70	4.9	9.00

*Values were obtained from models adjusted for age. LAP, BMI, WHpR and WHtR were all evaluated in log-scale.

BMI, body mass index; FPG, Fasting plasma glucose; LAP, lipid accumulation product; 2h-PCPG, 2 hour post challenge plasma glucose; WHpR, waist to hip ratio; WHtR, waist to height ratio.

Excluding older men, the LAP consistently explained more variations either in baseline or in future levels of FPG and 2h-PCPG than did BMI. LAP, however, ranked differently in relation to WHpR or WHtR, with no uniform pattern being observed.

### Diabetic status

**Figure 2:**
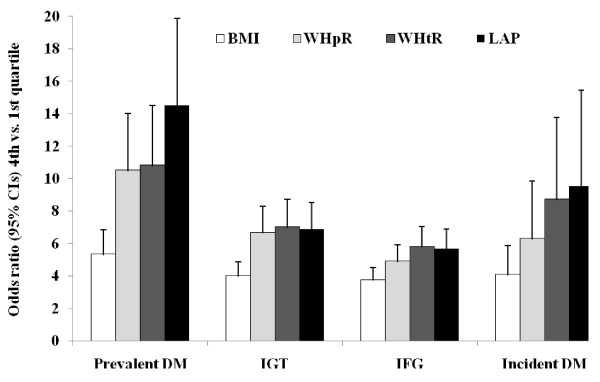
**Fourth vs. first quartiles of LAP versus BMI, WHpR, and WHtR, for identification of prevalent DM, IFG, and IGT, and prediction of incident DM**. BMI, body mass index; DM, diabetes mellitus; IFG, impaired fasting plasma glucose; IGT, impaired glucose tolerance; LAP, lipid accumulation product; WHpR, waist to hip ratio; WHtR, waist to height ratio.

As is depicted in figure 2, LAP was superior to BMI in identifying prevalent diabetes (P < 0.001); but not much so as compared to WHpR (P = 0.015) and WHtR (P = 0.014), considering the multiplicity of inference. Odds ratios (4^th ^vs. 1^st ^quartile) of LAP for IGT, IFG and incident diabetes were greater than those of BMI (Ps < 0.001), again LAP was found to be similar to WHpR (P_IGT _= 0.273, P_IFG _= 0.072, and P_Incident diabetes _= 0.220) and WHtR (P_IGT _= 0.563, P_IFG _= 0.470, and P_Incident diabetes _= 0.971).

The odds ratios for IGT, IFG, prevalent and incident diabetes corresponding to a 1 standard deviation increment in the baseline levels (log-scale) of LAP, BMI, WHpR, and WHtR estimated separately for each variable are presented in Table 3 and 4. The paired homogeneity test adjusted for baseline MAP and family history of diabetes indicated that odds ratios (size of risk) of LAP for incident diabetes did not differ from those for WHpR or WHtR. All condition indices were smaller than 5 and all VIFs were smaller than 4.

**Table 3:** Prediction of IFG and IGT by LAP, BMI, WHpR, and WHtR.

	**IFG**		**IGT**	
		
	**OR (95%CIs)^§^**	**AROC†**	**OR (95%CIs)^§^**	**AROC†**
		
Men				
20-49				
BMI	1.6 (1.3-2.0)	0.68	1.6 (1.3-2.1)*	0.69
WHpR	2.2 (1.7-2.9)	0.73	3.1 (2.2-4.2)	0.74
WHtR	2.0 (1.6-2.5)	0.72	2.3 (1.8-3.0)	0.73
LAP	2.0 (1.7-2.5)	0.73	2.3 (1.8-3.0)	0.75
P for trend	-	<0.001	-	<0.001
≥ 50				
BMI	1.4 (1.2-1.7)	0.65	1.5 (1.2-1.8)	0.61
WHpR	1.6 (1.3-2.0)	0.66	1.7 (1.4-2.2)	0.61
WHtR	1.6 (1.3-2.0)	0.65	1.6 (1.3-1.9)	0.61
LAP	1.4 (1.2-1.7)	0.70	2.2 (1.8-2.8)	0.66
P for trend	-	<0.001	-	<0.001
Women				
20-49				
BMI	1.4 (1.3-1.6)*	0.76	1.7 (1.4-2.0)*	0.75
WHpR	1.7 (1.4-1.9)*	0.78	2.3 (1.9-2.9)*	0.78
WHtR	1.7 (1.5-1.9)	0.79	2.3 (1.9-2.7)*	0.79
LAP	2.0 (1.7-2.3)	0.81	3.2 (2.6-4.1)	0.81
P for trend	-	<0.001	-	<0.001
≥ 50				
BMI	1.1 (1.0-1.3)	0.64	1.3 (1.1-1.5)*	0.64
WHpR	1.1 (0.9-1.3)*	0.68	1.6 (1.4-2.0)*	0.66
WHtR	1.2 (1.1-1.5)	0.67	1.7 (1.4-2.0)	0.66
LAP	1.5 (1.2-1.9)	0.71	2.6 (2.1-3.4)	0.71
P for trend	-	<0.001	-	<0.001

AROC, area under the receiver operating characteristic curve, BMI, body mass index; DM, diabetes mellitus; IFG, impaired fasting plasma glucose; IGT, impaired glucose tolerance; LAP, lipid accumulation product; OR, odds ratio; WHpR, waist to hip ratio; WHtR, waist to height ratio.

§The odds ratios (unadjusted) of incident and prevalent DM, IFG, and IGT were estimated using logistic regression analysis corresponding to a 1 standard deviation (log-scale) unit increment in the baseline LAP, BMI, WHpR, and WHtR.

*Paired homogeneity tests showed significant difference (P < 0.05). For paired homogeneity test LAP and one of anthropometric measures were fitted simultaneously in the same age- and sex-specific logistic regression model with adjustment for family history of diabetes and mean arterial pressure.

† The AROCs were computed for the sex- and age-specific models adjusted for mean arterial blood pressure and family history of diabetes.

**Table 4:** Prediction of incident and prevalent DM by LAP, BMI, WHpR, and WHtR.

	**Prevalent DM**		**Incident DM**	
		
	**OR (95%CIs)^§^**	**AROC†**	**OR (95%CIs)^§^**	**AROC†**
		
Men				
20-49				
BMI	1.3 (1.1-1.5) *	0.70	1.3 (0.9-1.8)*	0.66
WHpR	1.7 (1.4-2.1)	0.74	1.7 (1.0-2.7)	0.67
WHtR	1.5 (1.3-1.8)	0.74	1.4 (1.0-2.1)	0.66
LAP	1.4 (1.2-1.6)	0.75	1.7 (1.2-2.5)	0.71
P for trend	-	0.157	-	0.051
≥ 50				
BMI	1.6 (1.3-1.9) *	0.76	1.5 (1.0-2.2)	0.69
WHpR	1.6 (1.3-1.9) *	0.78	1.5 (0.9-2.4)	0.70
WHtR	1.6 (1.3-1.9) *	0.79	1.5 (1.0-2.3)	0.69
LAP	1.5 (1.3-1.8)	0.81	1.7 (1.1-2.6)	0.71
P for trend	-	0.005	-	0.492
Women				
20-49				
BMI	1.6 (1.5-1.9)*	0.76	1.9 (1.5-2.4)	0.76
WHpR	1.8 (1.6-2.1)*	0.78	2.2 (1.7-2.9)	0.77
WHtR	1.9 (1.3-2.1)*	0.79	2.3 (1.8-3.0)	0.79
LAP	2.1 (1.8-2.5)	0.81	2.6 (1.9-3.6)	0.78
P for trend	-	<0.001	-	0.012
≥ 50				
BMI	1.3 (1.1-1.4)*	0.65	1.5 (1.1-2.1)	0.63
WHpR	1.1 (1.0-1.3)*	0.68	1.6 (1.1-2.3)	0.64
WHtR	1.3 (1.1-1.5)*	0.68	1.9 (1.3-2.8)	0.65
LAP	1.5 (1.3-1.8)	0.72	2.1 (1.3-3.3)	0.65
P for trend	-	<0.001	-	0.567

AROC, area under the receiver operating characteristic curve, BMI, body mass index; DM, diabetes mellitus; IFG, impaired fasting plasma glucose; IGT, impaired glucose tolerance; LAP, lipid accumulation product; OR, odds ratio; WHpR, waist to hip ratio; WHtR, waist to height ratio.

§The odds ratios (unadjusted) of incident and prevalent DM, IFG, and IGT were estimated using logistic regression analysis corresponding to a 1 standard deviation (log-scale) unit increment in the baseline LAP, BMI, WHpR, and WHtR.

*Paired homogeneity tests showed significant difference (P < 0.05). For paired homogeneity test LAP and one of anthropometric measures were fitted simultaneously in the same age- and sex-specific logistic regression model with adjustment for family history of diabetes and mean arterial pressure.

† The AROCs were computed for the sex- and age-specific models adjusted for mean arterial blood pressure and family history of diabetes.

The difference in discriminatory capacity of the predictive age- and sex-specific models incorporating family history of diabetes, MAP, and either LAP, BMI, WHpR, or WHtR is presented in Table 3 and 4. AROCs obtained from competing models for identifying prevalent diabetes, IFG, and IGT and also incident diabetes. The discriminatory capacities in older participants were lower than in younger ones. LAP was, generally, superior to anthropometric variables in discriminating prevalent diabetes, IFG, and IGT. The discriminatory capacity of LAP for incident diabetes, however, was similar to those of BMI, WHpR, and WHtR.

## Discussion

Using data from a population based study, we extended the superiority in identifying prevalent diabetes of LAP over BMI, reported by Kahn [1], and showed that LAP had higher ORs for prevalent diabetes than did WHpR and WHtR; this superiority, however, was not observed in all subgroups. For different outcomes (IFG, IGT, prevalent or incident diabetes), no clinically significant difference was observed between discriminatory capacities of LAP, BMI, WHpR, or WHtR. WHtR, WHpR, and BMI showed as strong associations with incident diabetes as did LAP, except in young men. We observed that LAP was superior to BMI, and approximately equal to WHtR and WHpR for predicting FPG and 2h-PCPG levels.

The effect of LAP and other measures of adiposity on plasma glucose levels and prevalent and incident diabetes were moderated by age and sex, the finding that was also reported by previous studies [19]. It has been demonstrated that the variation in visceral adipose tissue accumulation can explain a significant proportion of the sex- as well as age-related differences in the metabolic risk profile [20]. Assessing risk in older patients is challenging since traditional risk factors are less predictive in older versus middle-age populations. Evidence suggests that obesity in the elderly may not be associated with the same risks as in younger individuals, and in certain aspects, can even be protective [21]. Age-dependent weight loss in later life with stable or increasing adiposity is characterized by a redistribution of the fat mass that favors enhanced visceral and ectopic adipose tissue accumulation [22]. Older individuals are generally shorter than younger individuals because of shrinkage of the spine due to vertebral bone loss, kyphosis, and scoliosis. The BMI of older individuals, hence, may be overestimated [23]. In the Rosetta Study, however, older adults had, on average, more fat than younger adults at any given BMI [24]. The distribution of body fat changes with age and relatively more fat accumulates in the abdomen and less fat at the extremities [25]. For a given WC, visceral fat has been shown to be higher in older individuals compared with younger individuals [26], suggesting that absolute levels of WC should be interpreted differently in younger and older persons. We hypothesized that the aging modifies the association of the anthropometric measures of adiposity and LAP with visceral and ectopic adipose tissue accumulation, which are thought of as mediators of the effect of adiposity on FPG or 2h-PCPG. Decreased insulin sensitivity at the cellular level is also a natural consequence of aging [22] that can, independent of adiposity, subject the elderly population to the higher risk of developing diabetes; and therefore affect the association of measures of adiposity with diabetes.

Different modalities have been used to characterize the relationship between visceral fat and diabetes. Imaging techniques that directly quantify the visceral adipose tissue and subcutaneous adipose tissue are costly and invasive; therefore, anthropometric measurements are more commonly utilized. LAP, has been reported to offer an inexpensive and non invasive tool to estimate total body lipid accumulation in comparison with sophisticated imaging methods for estimating the lipid burden or uptake in isolated tissues [1]. We observed, however, that if LAP is to be used for predicting diabetes, it might not be superior to WHtR or WHpR. The quantity of intra-abdominal fat being strongly related to the metabolic disorders is the basis for suggestions regarding the superiority of anthropometric measures that describe central fat distribution to general measures of obesity, with respect to the prediction of diabetes. Previous studies and most recently Kahn et al. have shown that height is an independent predictor of diabetes [2,27,28]. Hip circumference has also been previously reported to be independently and inversely associated with diabetes [29]. So the WHpR and WHtR may represent joint risk related to WC and hip and height, respectively. We have previously shown that WHtR is superior to the other anthropometric measures of obesity in predicting diabetes [30]. Anthropometric measures of central adiposity and general obesity rank variably in predicting diabetes in different ethnic populations [31]. Some studies recently compared different anthropometric measures in terms of their ability to predict diabetes and to determine whether predictive ability was modified by ethnicity. They observed that measures of central and overall adiposity predicted diabetes to a similar degree except for slight superiority for WHtR [32-34].

We have previously shown, in a cross sectional study, that TGs is independently associated with undiagnosed diabetes [35]. In the current study LAP was shown to be better at identifying prevalent diabetes than at predicting incident diabetes. This means that by the time one would expect the LAP to be elevated the elevated glucose levels might have been already attained; the finding that supports the view that diabetes is not merely impairment in glucose regulation, rather the complex metabolic consequences of accumulating ectopic lipids or hepatic fat [1-4].

We observed that anthropometric measures of adiposity as well as LAP explained more variations in 2h-PCPG levels than in FPG levels. The release of free fatty acids from visceral fat into the portal vein that directly leads to the liver may cause reduced hepatic insulin clearance, increased gluconeogenesis and increased dyslipidaemia [23] that manifests as increased 2h-PCPG. Aging and genetic predisposition (for which we were not able to control) contribute to β-cell dysfunction; the chronic glucotoxic and lipotoxic effects on FPG of the insulin-resistant state in obesity, might have been confounded by the genetic factor(s) [22].

The LAP, WHpR, and WHpR being more strongly associated with 2h-PCPG than with FPG, can potentially make them practical tools to be used for selecting those with normal FPG levels in whom testing 2h-PCPG may be of benefit. Our findings indicate that the predictive value of the LAP and WHpR or WHtR were similar. The message is important since WHpR and WHtR are easier to measure than is LAP; a major obstacle for many people could be the requirement of a venipuncture to obtain TGs levels in a fasting state for obtaining LAP levels [9].

## Conclusions

Although LAP showed some superiority over BMI in predicting FPG and 2h-PCPG and identifying prevalent diabetes; BMI, WHpR, WHtR, and LAP were relatively of the same capability for predicting diabetes, with no consistent superiority of one over the others across age- and sex-groups. Although individuals with high LAP had an increased risk of diabetes as compared with those with low LAP, the increment in the AROC of the models incorporating LAP over the model with BMI, WHtR, and WHpR was negligible. Hence, it may not be plausible to increase the resources needed for testing TGs levels.

## Competing interests

The authors declare that they have no competing interests.

## Authors' contributions

MB designed the study, performed the statistical analysis, and drafted the manuscript. FH and FA revised the manuscript critically for important intellectual content. All authors read and approved the final manuscript.
